# Pregnant women’s knowledge of weight, weight gain, complications of obesity and weight management strategies in pregnancy

**DOI:** 10.1186/1756-0500-6-278

**Published:** 2013-07-18

**Authors:** Alexis Shub, Emily Y-S Huning, Karen J Campbell, Elizabeth A McCarthy

**Affiliations:** 1 Department of Obstetrics and Gynaecology, University of Melbourne, Perinatal Centre, 3rd Floor, Mercy Hospital for Women, 163 Studley Road, Heidelberg, Victoria, 3078, Australia; 2 Centre for Physical Activity and Nutrition Research, School of Exercise and Nutrition Sciences, Deakin University (KC), Geelong, Melbourne, Australia

**Keywords:** Overweight, Obesity, Pregnancy, Body mass index, Weight gain, Knowledge, Complications

## Abstract

**Background:**

Obesity is increasingly common in the obstetric population. Maternal obesity and excess gestational weight gain (GWG) are associated with increased perinatal risk. There is limited published data demonstrating the level of pregnant women’s knowledge regarding these problems, their consequences and management strategies.

We aimed to assess the level of knowledge of pregnant women regarding: (i) their own weight and body mass index (BMI) category, (ii) awareness of guidelines for GWG, (iii) concordance of women’s own expectations with guidelines, (iv) knowledge of complications associated with excess GWG, and (v) knowledge of safe weight management strategies in pregnancy.

**Methods:**

364 pregnant women from a single center university hospital antenatal clinic were interviewed by an obstetric registrar. The women in this convenience sample were asked to identify their weight category, their understanding of the complications of obesity and excessive GWG in pregnancy and safe and/or effective weight management strategies in pregnancy.

**Results:**

Nearly half (47.8%) of the study population were overweight or obese. 74% of obese women underestimated their BMI category. 64% of obese women and 40% of overweight women overestimated their recommended GWG. Women’s knowledge of the specific risks associated with excess GWG or maternal obesity was poor. Women also reported many incorrect beliefs about safe weight management in pregnancy.

**Conclusions:**

Many pregnant women have poor knowledge about obesity, GWG, their consequences and management strategies. Bridging this knowledge gap is an important step towards improving perinatal outcomes for all pregnant women, especially those who enter pregnancy overweight or obese.

## Background

Overweight and obesity are common problems with an increasing worldwide incidence [1]. Recent Australian data showed that 50% of pregnant women were overweight or obese and in the United States 36% of women were obese [2,3].

Maternal obesity and excessive gestational weight gain (GWG) have well recognized associations with pre-eclampsia, gestational diabetes mellitus (GDM), instrumental or operative delivery, failed induction, fetal macrosomia, neonatal hypoglycaemia, perinatal mortality and infant and childhood obesity [4-7]. In addition, maternal obesity is the single most common modifiable factor in stillbirth in the developed world [8].

There is limited published data assessing the relationship between a woman’s actual and perceived Body Mass Index (BMI) in pregnancy, and the effect this has on GWG. It has been demonstrated that overweight and obese pregnant women are less likely than women of normal weight to correctly assess their own BMI [9], and that overweight women who underestimate their BMI are more likely to gain excess weight in pregnancy [10].

In addition to assessing pregnant women’s accuracy in estimating their own BMI, this study aimed to describe pregnant women’s awareness of GWG guidelines; knowledge of safe/effective methods of weight management in pregnancy and; awareness of complications of obesity and excess GWG in pregnancy. We aimed to determine if women’s knowledge was influenced by factors including age, antenatal care provider or education from a dietitian. A greater understanding of the expectations and levels of knowledge of pregnant women regarding these factors will allow us to better design programs to educate and assist women in achieving appropriate GWG.

## Methods

The study was approved by the Mercy Hospital for Women Human Research Ethics Committee. Participants were recruited by convenience sampling in the waiting room of an urban, university affiliated, tertiary maternity hospital between June and August 2010. The hospital has approximately 5000 deliveries per year with a wide mix of ethnicities represented. The sample size for this unfunded, descriptive study was limited by pragmatic considerations. After 2 months recruiting, 364 women had consented to be involved in the study.

**Figure 1:**
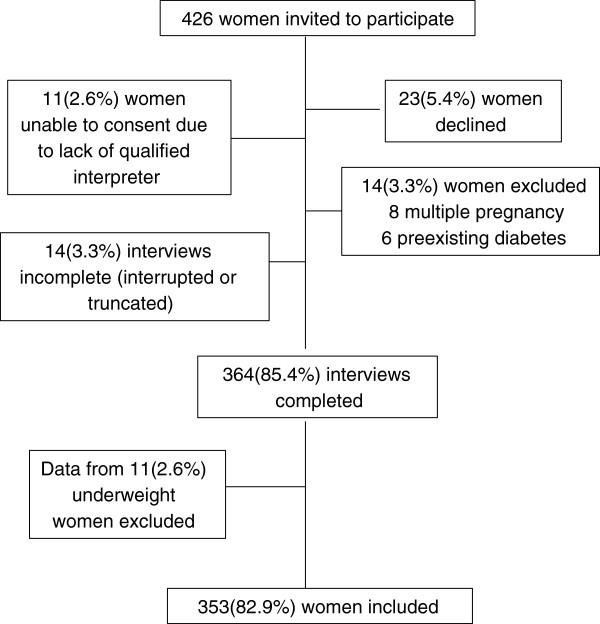
Subject participation in interview process.

Informed consent was obtained from each participant prior to commencement of the interview. Women were excluded from the study if they had a multiple pregnancy, diabetes mellitus prior to pregnancy or if they were non-English speaking without an appropriately qualified interpreter present. Figure 1 shows the flow of participants through the study. Twenty five women were ineligible to participate. Eleven women were underweight and due to the small numbers were excluded from further analysis.

Pregnancy weight and height were self-reported from early pregnancy, except where women did not know their weight or height and thus were weighed or measured by the researcher (EH) [11].

A 40 item questionnaire was developed by 2 of the authors (EH and AS) in conjunction with other obstetricians, midwives and dietitians and refined after piloting. Women were asked simple demographic information, then to identify themselves as being underweight, normal weight, overweight or very overweight. They were asked what they thought was the best pregnancy weight gain was for themselves. They were then asked if they believed that obesity in pregnancy or excess GWG were associated with problems for mother or for baby, and women who had replied positively were asked to list those problems. They were also asked whether a number of dietary practices were safe ways to manage weight gain in pregnancy (see Additional file 1).

The interviewer (EH), an obstetric registrar not involved in providing antenatal care to study participants at the time of the study, transcribed participants’ responses contemporaneously with pen and paper and later entered paper based form responses into a spreadsheet. The Institute of Medicine (IOM) guidelines for weight gain in pregnancy [12] and the Australian guide to healthy eating [13] formed the basis of “expert opinion” against which participant opinions were judged to be correct or incorrect. A one-week test-retest assessment of question reliability in a separate sample of 20 women demonstrated 100% agreement for 23 of 40 questions with no question having less than 65% agreement between test and re-test. Of 15 items for which kappa could be calculated, the median was 0.634 with interquartile range 0.443 to 0.798, confirming acceptable reliability.

After completing the questionnaire, women were offered information on their own BMI and the guidelines for weight gain in pregnancy, and advised to discuss their personal care further with their obstetric care provider.

Statistical analysis was performed using PASW Statistics 18 (IBM, Armonk, New York). Normally distributed continuous data was described as means and standard deviations. Categorical data was described using proportions and compared using Chi squared tests. p < 0.05 was considered significant.

## Results

Of the 353 participants, 179 (50.7%) women were classified by BMI as being normal weight (BMI 20-24.9 kg/m^2)^, 99 (28.0%) were classified as overweight (BMI 25-29.9 kg/m^2^) and 75 (21.2%) were classified as obese (BMI ≥30). Of women defined as obese, 41 (54.7%) were classified as obesity class I (BMI 30.0-34.9 kg/m^2^), 20 (26.7%) were classified as obesity class II (BMI 35.0-39.9 kg/m^2^) and 14 (18.7%) were classified as morbidly obese (BMI ≥40.0 kg/m^2^). The maximum BMI was 60 kg/m^2^. 171(48.4%) of the women were nulliparous, 182 (51.6%) had a tertiary education, 312 (88.4%) had seen a doctor or midwife during the pregnancy and 25 (7.1%) of had been diagnosed with gestational diabetes, but 81 women had not yet been tested. The median and interquartile age was 31.1(28-35) years and median gestation at the time of the interview was 28 (20-36) weeks. Hospital birth statistics from the hospital data base the same year indicate that the convenience sample was similar to the general population regarding parity (46.5% nulliparous), maternal age 31.0 (28-35) years, and BMI (51.1% normal weight, 28.9% overweight and 20.3% obese).

Women’s perceptions of their own weight are presented in Table 1. The majority (86.6%) of normal weight women identified themselves as such, however nearly one in 10 considered themselves overweight. While around two-thirds of women accurately identified they were overweight, the remainder considered themselves to be normal weight. The majority of obese women considered themselves to be overweight and these women were the most likely to underestimate their own BMI category, with only 24% identifying themselves as very overweight.

**Table 1:** Accuracy of estimation of BMI by women in each weight category

		**Perceived BMI category**			
		**Underweight**	**Normal weight**	**Overweight**	**Very overweight**
Actual BMI category	Normal	8(4.5)	155(86.6)	16(8.9)	0(0)
	Overweight	0(0)	34(34.3)	65(65.7)	0(0)
	Obese	0(0)	5(6.7)	52(69.3)	18(24.0)

Data is n (%), p < 0.05.

Normal weight was defined as BMI 20-24.9 kg/m^2^, overweight BMI 25-29.9 kg/m^2^, obese BMI ≥30.

As shown in Table 2, misperceptions regarding appropriate weight gain in pregnancy were commonplace, with overweight or obese women being least accurate at estimating appropriate GWG. Those women who underestimated their own weight were more likely to overestimate optimal weight gain in pregnancy.

**Table 2:** Accuracy of estimation of recommended weight gain in pregnancy by women in each BMI category and perceived weight category

	**Estimation of recommended weight gain**				
	**Underestimate**	**Correct**	**Overestimate**	**Unable to estimate**	
Women classified according to BMI category					
Normal n = 179	56(31.3)	104(58.1)	13(7.3)	6(3.4)	p = 0.001
Overweight n = 99	7(7.1)	47(47.5)	40(40.4)	5(5.1)	
Obese n = 75	10(13.3)	16(21.3)	48(64.0)	1(1.3)	
Woman classified according to difference between actual and perceived weight category					
Underestimate n = 98	10(10.2)	23(23.5)	61(62.2)	4(4.1)	p = 0.01
Correct n = 239	60(25.1)	132(55.2)	39(16.3)	8(3.3)	
Overestimate n = 16	3(18.8)	12(75.0)	1(6.3)	0(.0)	

Data is n (%).

Ninety-four per cent of women believed that excess GWG or obesity would be associated with increased pregnancy complications, but their knowledge of the specific nature of these risks was poor. 27.8% of women identified preeclampsia or blood pressure problems, 51% identified gestational diabetes and 14.4% suggested postpartum weight retention (data not presented in tables). 71% of women suggested concerns such as back pain or difficulty moving. Less than 5% of women suggested caesarean section, operative delivery, preterm birth or postterm delivery as being related to maternal BMI or GWG. 72.8% believed that there could be neonatal complications from obesity or GWG and 18.4% suggested macrosomia. Less than 5% of women suggested any other neonatal complications including hypoglycaemia, jaundice special care nursery admission or increased perinatal mortality.

Study participants were also found to hold many incorrect beliefs about safe weight management in pregnancy, more than one third of women believed that eating an organic diet, drinking more fruit juice, not eating after 8 pm or choosing full fat dairy products were safe ways to manage weight gain in pregnancy (Table 3). No association was demonstrated between women’s BMI category, parity, care provider, age or educational status and their level of knowledge of maternal or neonatal complications or beliefs about diet in pregnancy (data not shown).

**Table 3:** Women’s beliefs about safe and effective management of weight gain in pregnancy

	**Expert opinion**	**Number (%) of participants answering correctly**
**Dietary behavior**		
Skip meals	No	348 (98.6)
Eat for two	No	309 (87.5)
Remove fat from meat	Yes	311 (88.1)
Finish everything on your plate	No	309 (87.5)
Stop eating after eight pm at night	No	222 (62.9)
**Dietary approaches**		
Choose low fat milk and dairy products	Yes	233 (66.0)
Eat less cakes and chocolate	Yes	339 (96.0)
Eat a gluten free diet	No	278 (78.8)
Drink less soft drink	Yes	350 (99.2)
Drink more fruit juice	No	239 (67.7)
Eat plenty of fruit and vegetables	Yes	353 (100)
Eat less take away foods	Yes	352 (99.7)
Eat less fried foods	Yes	349 (98.9)
Eat an Atkins/low carbohydrate diet	No	298 (84.4)
Drink soy milk instead of cows milk	No	298 (84.4)
Eat an organic diet	No	181 (51.2)
**Exercise**		
Exercise 3 or more times each week	Yes	332 (94.1)
Avoid exercise	No	352 (99.7)

Total 353 women.

The Institute of Medicine (IOM) guidelines for weight gain in pregnancy [12] and the Australian guide to healthy eating [13] formed the basis of “expert opinion”.

## Discussion

In this convenience sample of obstetric patients from a large metropolitan hospital we show frequent misclassification of BMI. Women predominantly underestimated their degree of overweight or obesity and overweight and obese pregnant women were more prone to inaccurate self-classification compared with normal weight women. Twenty-four per cent of obese pregnant women accurately classified their BMI in the current study which is similar to rates of 10% in Canada [14], 16% in Brisbane, Australia [15] and 30% in Canberra, Australia [16]. By comparison 87% of normal weight women in our sample were accurate in classifying their BMI, a finding similar to 89% in Brisbane [15] and 94% recorded in Canada [14]. BMI classification is similarly inaccurate in some studies of non-pregnant overweight and obese women [17] where only 16% of obese and 31% of overweight women correctly classified their BMI by matching themselves against standard silhouettes. Weight perception is generally more accurate in women than men [18,19]. It is possible that the increasing prevalence of obesity is changing the community’s perception of what is “normal”.

Previous studies have asked pregnant women what they actually eat. To our knowledge, our study is the first to characterize pregnant women’s beliefs about appropriate dietary approaches to achieve safe and effective management of weight gain in pregnancy, demonstrating that many pregnant women’s ideas of appropriate diet in pregnancy diverge from expert opinion. The diet questions were intentionally very simple. This was for 2 reasons, firstly to give participants confidence in answering questions about a sensitive topic, weight gain and diet, and to demonstrate the low level of knowledge for even simple information. Midwives and obstetric doctors should not assume that pregnant women are using safe strategies to avoid excess GWG. Inaccurate or unsafe ideas about ways to contain gestational weight may contribute to the increasing frequency of excess GWG. Avoiding excess GWG is important for women in the long term [20], is associated with better perinatal outcome [4,21-23] and appears to reduce inter-generational transmission of obesity [24] but is not usual. More than half of overweight pregnant women gain in excess of that recommended by the IOM [20,25,26] and this trend is increasing rather than abating [27,28].

We have also demonstrated that pregnant women have a low awareness of the perinatal complications associated with excess maternal weight [14,29]. Women may be more aware of personal long term health risks rather than of perinatal risks associated with obesity [14]. Better awareness of these complications may provide a motivating factor for women to maintain appropriate GWG in order to improve outcomes for their baby.

The strengths of this study include a large sample size, and demographic details which are similar to state data [30] suggesting that despite sampling at a tertiary, Australian, urban maternity hospital the findings may be generalisable to the wider population. In contrast, Gaudet’s Canadian study overrepresented older, nulliparous, tertiary educated pregnant women compared with population data [14]. A single researcher performed all of the interviews to exclude interobserver variation. An interview process may increase participation compared with written surveys, especially for women less confident in their written language abilities.

A weakness in our study was that we did not assess ethnicity and therefore cannot comment on any cultural or ethnic differences in knowledge or perception of obesity or GWG. Knowledge of prepregnancy weight may have also been useful to understand how weight gain in pregnancy was impacted by women’s understanding of GWG targets. We also used self reported height and weight but self-report of height and weight in pregnancy has been found to have a high correlation with measured height and weight in a large Australian sample [11]. A convenience sample was used, which increased study numbers, however the demographics of the sample closely matched that of the hospital population as described in the results above.

Other reasons why many women, especially those commencing pregnancy overweight or obese, find it difficult to contain GWG within recommended limits could include:

misperception and knowledge gaps for pregnant women and/or maternity caregivers [31-33], professional lack of confidence in being able to help overweight and obese people improve their weight [33,34], inequitable distribution of overweight and obesity such that socio-economic deprivation commonly co-exists and this impedes access to good quality food and safe exercise [35].

Ours is the first Australian survey to assess women’s knowledge of GWG goals in an era and setting where serial weighing in pregnancy is not supported by local guidelines [36]. Canadian survey respondents had a more accurate understanding of recommended GWG in their setting where serial weighing remains part of routine antenatal care [14]. A previous randomized controlled trial (RCT) from our institution supports serial self-weighing, particularly for overweight women, to achieve specific GWG goals [37]. This is being extended in a new RCT which includes obese as well as overweight women and is powered to demonstrate a clinically meaningful degree of improvement in obstetric complications and in which psychological benefit or detriment of serial weighing will also be addressed (Australian New Zealand Clinical Trial Registry number ACTRN12611000881932).

Regarding research implications, we did not explore sources of error leading to inaccurate self-classification of BMI, inappropriate GWG goals, inaccurate knowledge of obesity-related perinatal complications or safe methods to contain GWG. The Canadian survey found that maternity professionals were pregnant women’s most common source of information about weight in pregnancy but that 59.8% of women obtained information from the internet [14]. In other research obstetric care providers have been shown to lack skills and confidence in counselling women around weight management [10,38]. Future research into professional and lay sources of information, including mobile telephone and web-based social networking can help plan novel behavioural programmes, especially for women entering pregnancy obese or overweight. This is particularly important since a systematic review of studies using behavioral advice, diet and physical activity shows only inconsistent success in limiting GWG [39]. Outside of pregnancy, commercial weight loss programmes may be more successful than health professional led programmes [34].

## Conclusion

Obesity and excessive GWG are increasing problems in the obstetric population. Lack of knowledge of personal BMI, GWG targets limits and appropriate weight management strategies may limit the ability of women to address these issues successfully during their pregnancy.

## Abbreviations

BMI: Body mass index; IOM: Institute of medicine; GWG: Gestational weight gain; GDM: Gestational diabetes mellitus; RCT: Randomized controlled trial

## Competing interest

The authors declare they have no competing of interest.

## Authors’ contributions

AS and EH designed research; EH conducted research; AS analyzed data; AS, EM and KC wrote the paper; AS had primary responsibility for final content. All authors read and approved the final manuscript. The authors would like to acknowledge the assistance of Dr Carolyn J. Wicks with data entry and collation. All authors read and approved the final manuscript.

## Supplementary Material

Additional file 1 Questionnaire - Women’s knowledge of pregnancy weight gain. Click here for file
