# Supplementary material for: Pregnant women’s knowledge of weight, weight gain, complications of obesity and weight management strategies in pregnancy
Source: BMC Res Notes. 2013 Jul 18;6:278. doi: 10.1186/1756-0500-6-278 (PMC3726511; doi:10.1186/1756-0500-6-278)
Supplement: Additional file 1 — Questionnaire - Women’s knowledge of pregnancy weight gain. [file 1756-0500-6-278-S1.doc]

**Questionnaire - Women’s knowledge of pregnancy weight gain**

| 1^st^ hospital visit | Y / N | | | Seen midwife /Dr | | |
| --- | --- | --- | --- | --- | --- | --- |
| Gestation |  | | |  | | |
| If >28 Gest Dm | Y / N | | |  | | |
| diabetes | Y / N | | | EXCLUDE IF PREPREG DM | | |
| Seen dietician in this preg | Y / N | | |  | | |
| Singleton preg | Y / N | | | EXCLUDE IF MULTIPLE | | |
| Parity | Y / N | | |  | | |
| Age |  | | |  | | |
| education | <yr 12 / yr12 / tertiary / tafe or other | | | | | |
| Weight booking visit |  | | | |  | |
| Height |  | | | |  | |
| Perceived weight category | underweight | Healthy weight | | | overweight | Very overweight |
| What do you think is the best amount of weight to gain in pregnancy for someone of your weight? | ____________/ don’t know | | | | | |
| Do you think that too much weight gain in pregnancy or being overweight can increase problems in pregnancy for the mother? What sort of problems? | Yes / no / not sure | | PET, DM, macrosomia, CS, instrumental, preterm, post term, postpartum weight retention, difficulty breastfeeding,  Back pain, difficulty moving, fluid retent | | | |
| Do you think that too much weight gain in pregnancy or being overweight can increase problems in pregnancy for the baby ? What sort of problems? | Yes / no / not sure | | Macrosomia, birth trauma, hypoglycaemia, jaundice, childhood obesity, adult obesity/DM, perinatal mortality, SCN admission | | | |

| Which of these methods do you think are safe ways to manage weight gain in pregnancy? | |
| --- | --- |
| Have less soft drink | Yes/ No |
| Skip meals | Yes / No |
| Choose low fat milk and dairy products | Yes / No |
| Have less cakes and chocolate | Yes / No |
| Have a gluten free diet | Yes / No |
| Avoid exercise | Yes / No |
| Have more fruit juice | Yes / No |
| Eat plenty of fruit and vegetables | Yes / No |
| Exercise 3 or more times each week | Yes / No |
| To remind you - Which of these methods do you think are safe ways to manage weight gain in pregnancy? | |
| Eat for 2 | Yes / No |
| Stop eating after 8 at night | Yes / No |
| Remove fat from meat | Yes / No |
| Finish everything on your plate | Yes / No |
| Have less take away foods | Yes / No |
| Have less fried foods | Yes / No |
| Have an Atkins/low carbohydrate diet | Yes / No |
| Drink soy milk instead of cows milk | Yes / No |
| Have an organic diet | Yes / No |

Thank you for taking part – your BMI is ____ and recommended weight gain is ____
